# Coincidence of autosomal dominant polycystic kidney disease and Alport syndrome: a case report and literature review

**DOI:** 10.1007/s13730-025-01057-3

**Published:** 2026-01-20

**Authors:** Rui Liu, Fei Liu

**Affiliations:** 1 Department of Nephrology, Shulan (Hangzhou) Hospital Affiliated to Zhejiang Shuren University Shulan International Medical College, Hangzhou, 310022 Zhejiang China; 2 Department of Nephrology, The Children’s Hospital, Zhejiang University School of Medicine, National Clinical Research Center for Child Health, Hangzhou, 310000 Zhejiang China

**Keywords:** ADPKD, Alport syndrome, Genetic testing, Case report

## Abstract

Autosomal dominant polycystic kidney disease (ADPKD) is the most common genetic kidney disease, accounting for approximately 5% of kidney failure worldwide. While Alport syndrome (AS) is an inherited progressive disease that typically presents with microhematuria in childhood and subsequently progresses to hematuria, proteinuria, and progressive renal impairment. The coexistence of ADPKD and AS is rare. Here, we present a case of a child with ADPKD and AS who presented with bilateral renal cysts and hematuria. Furthermore, we described the clinical and genetic characteristics of the present case and summarized the above features of previously reported cases. A 4-year-old boy had persistent microscopic hematuria and intermittent gross hematuria for 1 year. During routine examinations, microscopic hematuria (++), bilateral renal cysts, and high-frequency hearing loss were discovered. Furthermore, His mother had microscopic hematuria and was later diagnosed with ADPKD. A pathogenic variant was found in *COL4A5* through genetic testing, while a variant of undermined significance (VUS) was discovered in *PKD1*. These clinical and genetic findings are consistent with the diagnosis of ADPKD and AS co-occurring. Enalapril was administered. A 2-year follow-up revealed that the patient had continuous microscopic hematuria and normal range renal function. We also reviewed four cases of ADPKD and AS coexisting. Moreover, hematuria and decreased renal function were the most common clinical features. They all had a positive family history. This disease has a poor prognosis, with three cases progressing to kidney failure. ADPKD and AS are often associated with hematuria, renal cysts, and decreased renal function. Comprehensive clinical information and early genetic testing is critical for diagnosis, prognostic stratification, and initiating targeted therapies to delay progression.

**Supplementary Information:**

The online version contains supplementary material available at 10.1007/s13730-025-01057-3.

## Introduction

The most prevalent monogenetic causes of chronic kidney disease are ADPKD and AS. ADPKD is an inherited multisystem disorder caused by mutations in the *PKD1*, *PKD2*, *DNAJB11*, and *GANAB* genes [1]. It is distinguished by progressive kidney enlargement. The symptoms of this disease begin in childhood, with hypertension, proteinuria, hematuria, and urinary concentration defects. AS is a progressive hereditary renal disease that causes sensorineural hearing loss and ocular abnormalities [2]. The coexistence of these two severe, inherited renal disorders is very rare. A systematic literature search was conducted in PubMed, EMBASE, and Cochrane Library (January 2000–December 2024) using keywords: (“ADPKD” OR “polycystic kidney disease”) AND (“Alport syndrome” OR “COL4A5” OR “COL4A3” OR “COL4A4”) AND (“coexistence” OR “co-occurrence”). We are aware of only four cases of patients who have both ADPKD and AS by literature searching. Herein, we present a case study of this coexistence and conduct a literature review.

## Case presentation

**Fig. 1:**
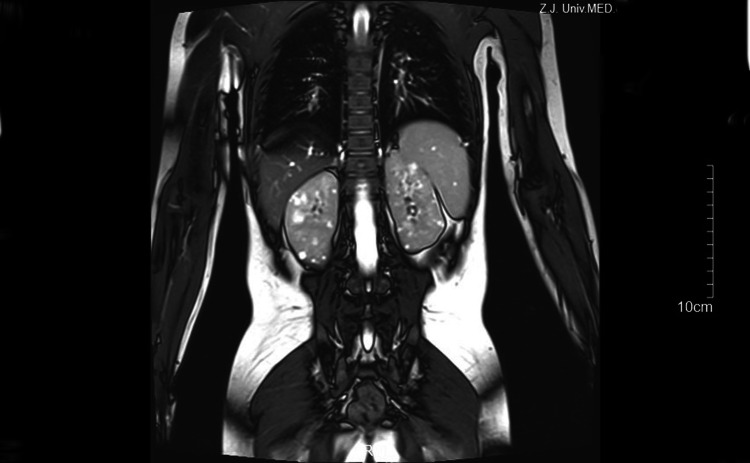
MRI of the bilateral kidney. Scattered multiple cysts can be seen on both sides of the kidney from MRI

A 4-year-old male presented to our department with a 1-year history of persistent microscopic hematuria and intermittent gross hematuria. We reviewed the medical history of the patient and discovered that he had never experienced edema, oliguria, and other renal symptoms. The birth history and the growth and development history were all normal. At the time of admission, his vital signs and other systemic examination findings were normal. After admission, urinalysis showed microhematuria but no proteinuria. His leukocyte and erythrocyte sedimentation rate (ESR) levels were not elevated, and his serum creatinine and blood urea nitrogen (BUN) levels remained normal. His antinuclear antibody (ANA) and antineutrophil cytoplasmic antibody (ANCA) panels were negative, and his complement and immunoglobulin levels were normal. Abdominal ultrasonography was performed, and bilateral renal cysts were discovered. This finding was confirmed by a kidney MRI (Fig. 1). Further investigation revealed high-frequency hearing loss. Ophthalmologic examination revealed no lenticonus, retinopathy, or other ocular abnormalities. We also learned that his mother had microscopic hematuria since childhood but no hearing loss, ocular abnormalities, or other extrarenal manifestations of ADPKD (e.g., hepatic cysts). Recent ultrasound confirmed bilateral renal cysts meeting Pei-Ravine criteria for ADPKD.

**Fig. 2:**
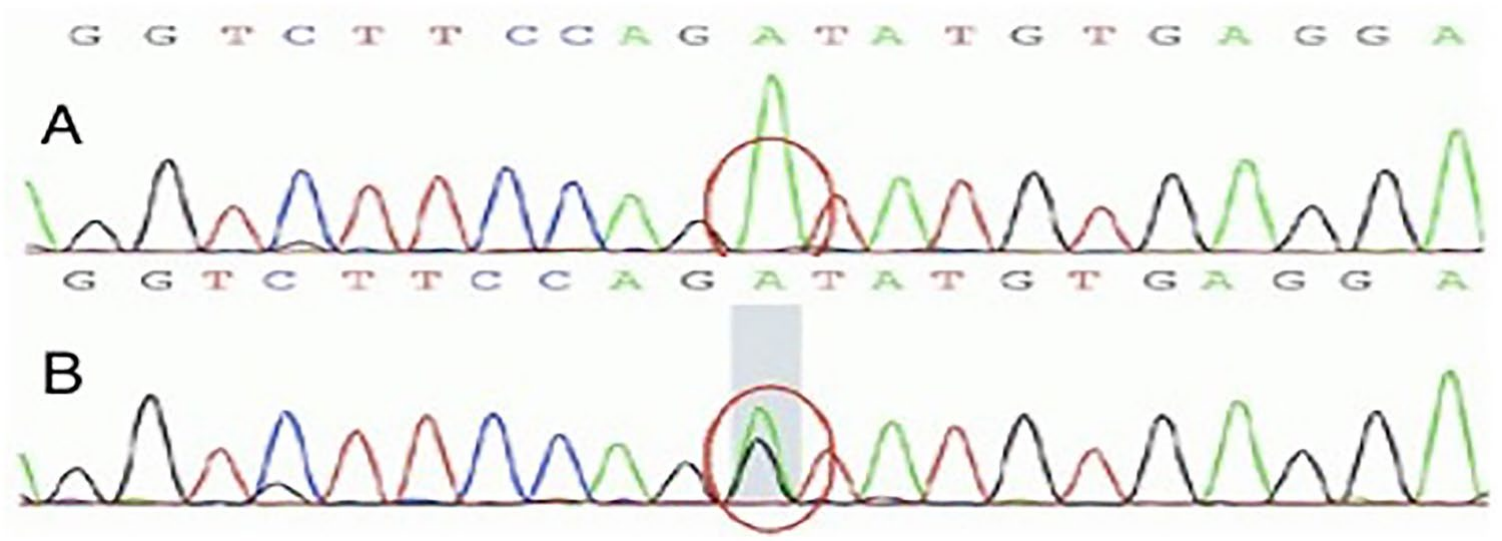
Verified the variant of *COL4A5* by Sanger sequencing. A hemizygous variation (c.2041+1G > A) in *COL4A5* (above). His mother was heterozygous for the same variation (below)

**Fig. 3:**
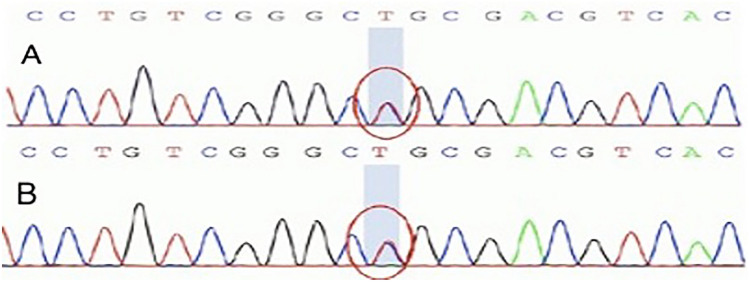
Verified the variant of *PKD1* by Sanger sequencing. A heterozygous variant (c.6070C > T; P. R2024C) was found in *PKD1* (above). His mother was heterozygous for the same variant (below)

The genetic testing was then performed. Genetic diagnosis counseling was conducted by a clinical nephrologist and genetic counselor. Social and psychological stress introduced by genetic diagnosis, as well as future financial and emotional burdens were discussed. Based on whole-exome sequencing (WES), we found a hemizygous variant (c.2041+1G > A) in COL4A5 (NM_033380), which was confirmed by Sanger sequencing (Fig. 2). This splice site mutation was predicted to influence splicing by software analysis (Human Splicing Finder). According to the American College of Medical Genetics (ACMG) interpretation guidelines, this mutation was classified as a pathogenic mutation. His mother had the same variant but was heterozygous for it. We also discovered a heterozygous variant (c.6070C > T; P. R2024C) in PKD1 (NM_001009944) (Fig. 3). In silico analyses (SIFT, Polyphen2, and Mutation Taster) predicted that this missense variant was pathogenic, which has been included in the Human Gene Mutation Database (HGMD), and it was classified as a VUS according to the ACMG interpretation guidelines. His mother was also heterozygous for the same variant. Notably, the recent B-ultrasound result of the mother (Supplementary Figure S1) indicated bilateral kidney enlargement and multiple cysts (more than 10 on each side, a maximum expansion of 5.5 × 5.5 cm), which provided one more piece of evidence to diagnose ADPKD in this child according to the Pei-Ravine criteria [3].

According to the guidelines and consensus, males with X-linked AS should carefully manage hypertension, proteinuria, and dyslipidemia. Even before the onset of proteinuria, treatment with angiotensin-converting enzyme (ACE) inhibitors could delay the onset of kidney failure and improve life expectancy [4–6]. The safety and efficacy of nephroprotective therapy with ramipril in children with AS has been investigated [7]. The main methods in treating pediatric ADPKD are regular monitoring of blood pressure and proteinuria and regular imaging to monitor cyst growth. Tolvaptan, a promising medication, could help to slow disease progression [8–10]. The safety and efficacy of tolvaptan in children/adolescents with ADPKD has also been investigated in randomized controlled trial, which support the activity of tolvaptan at the vasopressin V2 receptor in children and adolescents with ADPKD [11]. In our case, enalapril was prescribed during an outpatient follow-up. He was advised to have his blood pressure, urinalysis, renal function, and total kidney volume checked regularly. We followed up on the progress of this patient 2 years later. He had continuous microscopic hematuria and renal function that was within normal limits (Supplement Fig. S1). While his mother had microscopic hematuria, a little amount of proteinuria, and normal kidney function.

## Discussion

The most prevalent inherited kidney disease is ADPKD, while AS is the second most common monogenic cause of chronic kidney disease (CKD) after ADPKD. The coexistence of ADPKD and AS has been extremely rare. To date, only four cases have been reported (Table 1) [12–14].

**Table 1:** Previous reports of coexisting mutations. (Clinical and laboratory data were extracted at the time of genetic diagnosis.)

Publication	Age	Gender	Symptom	Mutations	Family history	Outcome
				*PKD1 COL4A5/A3*		
Phelan et al. [12]	19	M	Hematuria deafness	c.4884C > T; c.2605G > A p.G869A (*COL4A5*)	Mother Sister: PKD	Kidney failure at age 16, under native nephrectomy at age 17, waiting KT
Miao et al. [13]	46	M	Hematuria Flank pain	c.3903delc; c.2858G > T (*COL4A5)*	Mother: PKD	Kidney failure at age 45
	58	M	Hematuria	c.3903delc; c.2858G > T (*COL4A5*)	Father: PKD	Kidney failure at age 44
Ebner et al. [14]	17	M	Hematuria deafness	c.8017−3C > G c.3549dupG (*COL4A3*)	Mother: PKD	eGFR decreased to 65 ml/min/1.73 m^2^ at age 17

All these previous cases were male. The ages of the participants ranged from 17 to 58 years. Besides deafness and flank pain, hematuria was present in all patients. All these patients had a positive family history of PKD. Among them, two patients developed kidney failure in their 40 s, one case developed kidney failure at the age of 16, and one case revealed decreased eGFR at the age of 17. Three patients were on hemodialysis, and one was being treated with an ACE inhibitor and an Angiotensin II Receptor Blocker (ARB).

Three cases tested positive for the *COL4A5* mutation, which was first reported by Phelan et al. as a de novo event, while one tested positive for the *COL4A3* mutation. All these patients had a *PKD1* gene mutation. Miao et al. reported a case with a de novo *PKD1* frameshift mutation, while the case reported by Ebner et al. had a de novo pathogenic *PKD1* mutation.

Phelan reported that one patient first developed kidney failure and then was diagnosed with sensorineural hearing loss and that NGS revealed coexisting ADPKD and AS. His mother and sister have ADPKD as well, but their renal function is normal [12]. Miao reported two patients in a large Chinese family with PKD coincidence of AS (diagnosed genetically). These two patients all had typical PKD, which was initially thought to be the cause of kidney failure. In this family, a novel frameshift *PKD1* mutation was found [13]. Ebner demonstrated a male patient with ADPKD and AS from a family with both ADPKD and AS [14].

Our case also shows the extremely uncommon coexistence of these two severe, inherited renal diseases. Based on clinical presentation and imaging findings, the first question is whether hematuria and renal cysts can be explained by monism or dualism. The hearing loss and family history of hematuria revealed new information. These findings point to the possibility of a genetic disease that could explain both conditions, such as AS. Multiple renal cysts are typically associated with multi-cystic dysplastic kidney (MCDK) or PKD, with ADPKD being the most common genetic kidney disorder. Given the fact that the boy had multiple renal cysts, a renal biopsy, which plays an important role in differential diagnosis, kidney biopsy was not performed due to the patient’s young age, multiple bilateral cysts (increasing bleeding risk), and definitive genetic diagnosis. The first line of defense against the coexistence of hematuria and multiple renal cysts is genetic testing. Especially since this patient has a tentative diagnosis of AS, it is necessary to investigate the possibility of other genetic mutations associated with renal cysts. The diagnosis of AS of this child was based on hematuria, hearing loss, positive family history, and a pathogenic variant, while the diagnosis of ADPKD was mainly based on clinical information, as genetic testing only found a VUS in *PKD1*. Other reported cases had the same experience: patients were consulted for hematuria or renal cysts, with a positive family history, and NGS helped confirm the coexistence of PKD and AS.

As we all know, AS is conventionally diagnosed pathologically, but recent advances in comprehensive genetic analysis have enabled genetic testing to be used as a first-line diagnosis for AS. While ADPKD imaging-based diagnostic criteria need a positive family history (lacking in 10%–25% of the patients), genetic diagnostics are rarely performed. when the coincidence is suspected, genetic testing should be done.

AS is induced by mutations in any of three type IV collagen genes: *COL4A3*, *COL4A4*, or *COL4A5*. Some papers have been published on the relationship between collagen IV gene mutation and renal cysts: *COL4A1* was identified as a candidate gene in hereditary angiopathy with nephropathy, aneurysms, and muscle cramps (HANAC) syndrome with AD hematuria, cystic kidney disease, intracranial aneurysms, and muscle cramps [15]. In thin basement membrane disease (TBMD) patients (with or without collagen IV gene mutations), a high-prevalence of multi-cystic kidneys was discovered [16]. Collagen IV gene mutations have been reported in patients with ADPKD with an underlying *PKD1* or *PKD2* mutation [17]. Furthermore, patients who identified with *PKD2* and *COL4A1* mutations developed kidney failure earlier than those with monogenic disorders [18]. The cause and trigger of this coincidence are unknown; the inherent association, such as a potential aggravating effect of these two gene mutations, needs further investigation.

Furthermore, ADPKD is characterized by the progressive development and cystic dilation of the renal tubules, with nearly half of cases ultimately leading to kidney failure by the age of 60. In addition, nearly all affected X-linked AS males have decreased kidney function, which will lead to kidney failure as early as the second decade of life [19]. Therefore, the outcome of this coincidence is not favorable. Three previously reported patients were diagnosed with kidney failure when they first came in for a consultation, and one of them had decreased renal function. In our case, the renal function of this child was within the normal range, which was related to the early stage of ADPKD and AS. Considering the outcomes of previous cases, our patient is likely to develop kidney failure in near future. Due to early detection and diagnosis, some interventions and prescriptions may slow the disease’s progress.

Overall, our case and literature demonstrate the coexistence of ADPKD and AS. When a patient with ADPKD has other out-renal symptoms and positive inherited disease history, other gene mutations should be suspected. Given the poor prognosis, the coexistence of ADPKD and AS should be treated with the ACE inhibitor, tolvaptan, and other treatments to help delay the progression. More clinical case reports and further investigation are needed to better understand this disease.

## Supplementary Information

Below is the link to the electronic supplementary material.

Supplementary file 1. Fig S1. Timeline diagram of the patient (PDF 15 KB)
