# Supplementary figures and images for: Coincidence of autosomal dominant polycystic kidney disease and Alport syndrome: a case report and literature review
Source: CEN Case Rep. 2026 Jan 20;15(1):24. doi: 10.1007/s13730-025-01057-3 (PMC12819896; doi:10.1007/s13730-025-01057-3)

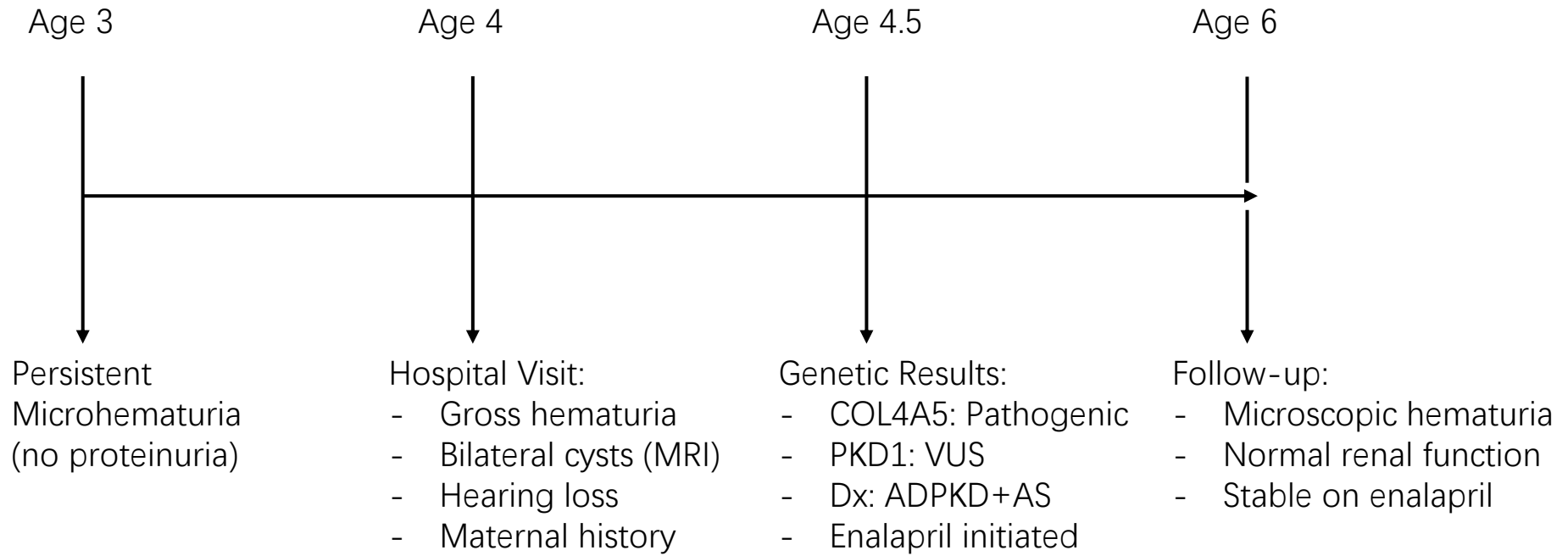

Supplement: Supplementary file 1 — Fig S1. Timeline diagram of the patient (PDF 15 KB) [file 13730_2025_1057_MOESM1_ESM.pdf]
